# Comparison of the Objective Severity and the Esthetic Perception of Nail Symptoms in Psoriasis

**DOI:** 10.1159/000521930

**Published:** 2022-02-14

**Authors:** Júlia Szebényi, Péter Oláh, Rolland Gyulai

**Affiliations:** ^a^Department of Dermatology, Venereology and Oncodermatology, University of Pécs Medical School, Clinical Centre, Pécs, Hungary; ^b^Department of Dermatology, University Hospital Duesseldorf, Medical Faculty, Heinrich-Heine-University, Duesseldorf, Germany

**Keywords:** Nail psoriasis, Nail Psoriasis Severity Index, Subjective assessment of nail psoriasis

## Abstract

**Introduction:**

Nail changes are frequent in psoriasis, and the negative impact of nail psoriasis on patients' quality of life is well known. No data are available however about the association of the objective severity of nail psoriasis and the subjective perception of these symptoms. The purpose of this study was to determine the correlation between the severity of psoriatic nail changes (as determined by the Nail Psoriasis Severity Index [NAPSI]) and the esthetic assessment of nail psoriasis.

**Methods:**

Participants (general population and psoriasis patients) were asked to rate 19 nail images (including psoriatic and healthy nails) on a 0–10 scale, based on how disturbing they considered them esthetically. Objective severity (NAPSI) scores of nails were compared to the subjective evaluation values.

**Results:**

Nail symptom severity correlated well with the subjective scores. However, while nails with low (0) and high (6–8) NAPSI values received consistent subjective scores, the esthetic perception of nails with moderate NAPSI scores was rather heterogeneous. The age of the respondents showed robust positive correlation with the subjective assessment of nail symptoms both within the psoriatic and the general population.

**Discussion:**

Gender, the presence of psoriasis, or medical education had no significant influence on the esthetic assessment of psoriatic nail changes.

## Introduction

Psoriasis is a common chronic inflammatory skin disease, affecting 2.0–6.5% of the European population [1]. While the prevalence of nail symptoms among psoriasis patients is up to 80% [2, 3], it is frequently disregarded in clinical practice. Nail psoriasis has been shown to be a predictive marker for psoriatic arthritis [4]. Treatment of nail psoriasis still represents a challenge for both the patients and physicians. Recently, new systemic anti-psoriatic therapies showed excellent results for treating nail symptoms as well [5].

While nail psoriasis severity scoring systems (e.g., Nail Psoriasis Severity Index [NAPSI] and modified NAPSI [mNAPSI]) focus on the objective physical alterations caused by the disease, less data are available about the subjective distress elicited by the nail symptoms. According to de Jong et al. [6], more than half of the patients suffer from pain caused by the nail changes, and a similarly large group of patients is restricted in their daily activities or profession. Some studies have also explored the association between nail psoriasis and impairment of health-related quality of life (HRQoL) [7], daily house-work routine, and social life [8]. Although DLQI was developed for assessing skin-related quality of life (no question concerns nail), some authors applied it for the assessment of nail psoriasis HRQoL [9, 10]. Ortonne et al. [11] also developed and validated a HRQoL scale specifically for nail psoriasis, the Nail Psoriasis Quality of Life (NPQoL) [11].

Interestingly, data regarding the cosmetic or esthetic concerns related to nail psoriasis are even scarcer. According to de Jong et al. [6], 93.3% of patients experience cosmetic problems related to their psoriatic nails. However, whether nail psoriasis severity is proportional to the esthetic problems-, and whether there are differences (e.g., age, gender, or the presence of psoriasis) in the esthetic perception of nail symptoms have not been previously systematically addressed. Therefore, the purpose of this study was to determine the correlation between the objective severity (NAPSI) and the subjective, esthetic perception of nail psoriasis by psoriasis patients (with and without nail symptoms) and the general public. Furthermore, we assessed whether age, gender, or professional medical knowledge of the assessors would influence the result.

## Materials and Methods

A cross-sectional survey using an online questionnaire − shown in online Supplementary Material 1(for all online suppl. material, see www.karger.com/doi/10.1159/000521930) − was set up to investigate the differences of the subjective/esthetic evaluation of nail psoriasis between psoriasis patients and the general population. The online questionnaire was distributed among psoriasis patients (via a psoriasis patient organization) and a group of healthy volunteers (including medical students, medical workers, and nonmedical workers). Convenience samples were applied, and responses were collected anonymously.

Respondents were asked to specify their age, sex, medical history, and in case of psoriasis patients, the absence/presence of nail psoriasis and current or previous systemic anti-psoriatic drug administration (the latter information was used to characterize moderate to severe psoriasis). Participants were then asked to evaluate 19 nail photos subjectively and to score all images based on how disturbing they consider them, using numeric rating 0 (no esthetic disturbance) to 10 (maximal esthetic disturbance). Nail photos were chosen from the archives of the Department of Dermatology, Venereology and Oncodermatology, University of Pécs Medical School. Objective severity of nail psoriasis was determined by calculating the NAPSI scores (0–8 point original scoring was used) [12]. Nail photos were selected to represent all nail psoriasis severity grades: healthy and mild (NAPSI 0–3, *n* = 4), moderate (NAPSI 4–6, *n* = 10), and severe (NAPSI 7–8, *n* = 5). The nail photo sequence was randomly assigned. In some experiments, during statistical analysis and data visualization, in order to correlate the objective nail severity scores with the subjective scores, NAPSI values (0–8) were scaled to 0–10 (that is, NAPSI values were multiplied by 1.25).

Statistical analysis was carried out using Graphpad Prism v7 and R v3.5.1 programs. For between-group differences of two and more groups, Mann-Whitney U tests and Kruskal-Wallis tests were used, respectively. Spearman correlations and linear regression were calculated using the cor.test and lm R functions, respectively. For head-to-head comparisons in Table 1, objective NAPSI scores (range 0–8) were scaled to 0–10 range. *p* values <0.05 were considered significant.

This investigation was approved and controlled by the Regional Research Ethics Committee of the Medical Center, University of Pécs (approval no. 3280/a) in accordance with the ethical principles of the Declaration of Helsinki and with Good Clinical Practice as defined by the International Conference on Harmonization.

## Results

### Demographic Characteristics

Between October 10, 2015, and February 25, 2016, 362 psoriatic patients and healthy volunteers completed the questionnaire. All responses were evaluated for data quality by a member of the investigator team (J.S.). Data of 68 respondents were excluded from the analysis because respondents scored all photos equally or scored healthy nail photos as severely disturbing (8–10 points). Data of 28 medical workers were also excluded, due to low sample size and significant age heterogeneity within the group. Altogether, data of 106 psoriatic patients (29% of respondents) and 160 respondents from the general population (86 medical students and 74 non health-care workers) were included in our analysis.

Of the 266 respondents included in our analysis, 175 (66.0%) were female, and 91 (34.0%) were male. Mean age was 38.4 ± 16.7 years, and 14.2%, 57.6%, and 28.3% of participants were <30 years, between 30 and 60 years, and >60 years, respectively. Among psoriatic patients, 75.5% had nail psoriasis, and, based on the history of anti-psoriatic drug use, 28.3% had severe psoriasis shown in Table 2.

### Subjective Scoring of Nail Images

**Fig. 1:**
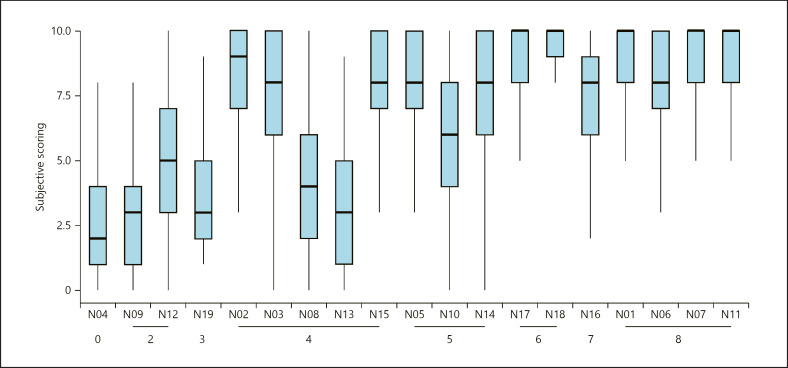
Subjective scoring (scale 0–10) of individual nail images. Boxplot center lines indicate the median value. *X*-axis numbering indicates the objective NAPSI score of respective nail images. Considerable subjective scoring heterogeneity within NAPSI categories 2, 4, and 5 indicate pronounced differences between objective and subjective scores.

The 19 nail images were scored by all respondents with a 0–10 score based on how disturbing the participant found the image esthetically shown in Figure 1 and Table 1. The lowest mean scores were given to N04 (2.64 ± 2.17) and N09 (3.1 ± 2.39). This was a healthy nail and a NAPSI 3 psoriatic nail, respectively. The highest score was ascribed to N18 (9.05 ± 1.68), a nail with NAPSI 6 score, and there were 4 more nails (N1, N7, N11, and N17, with NAPSI scores 8, 8, 8, and 6, respectively) with mean scores above 8.5. Overall, nail symptom severity, as assessed by the NAPSI score, correlated with the subjective scores. While nails with low (0) and high (6–8) NAPSI values received relatively consistent subjective scores, considerable subjective scoring heterogeneity was seen in case of nails with NAPSI values between 2 and 5 shown in Figure 1 and Table 1.

### Correlation of the Subjective Scoring of Nails and Age of Respondents

**Fig. 2:**
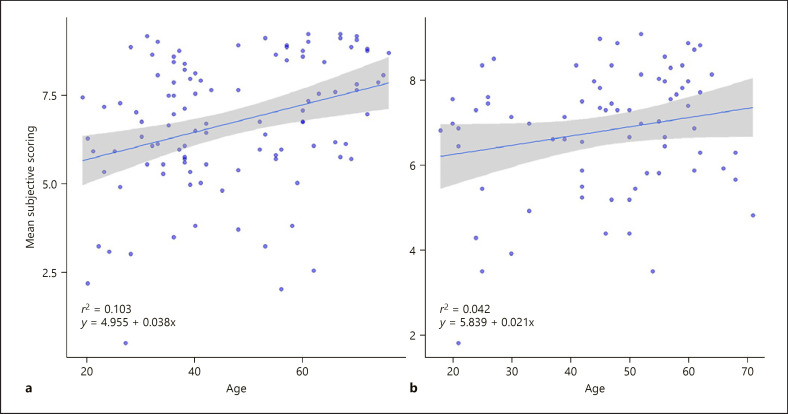
Linear regression of the mean subjective scoring of 19 nail images and participant age within psoriatic patients (**a**) and the general population (**b**). Each dot represents the score of a respondent. Dots may be slightly jittered to avoid overlaps. Age shows robust positive correlation with the subjective assessment of nail symptoms in both populations. Solid lines: line of best fit. Shaded area: 95% confidence interval.

Next, we compared the subjective scores of the 19 nail images with the age of the respondents. Age showed robust positive correlation with the subjective assessment of nail symptoms both within the psoriatic patients and the general population (*r*^2^ = 0.103, ŷ = 4.955 + 0.038x and *r*^2^ = 0.042, ŷ = 5.839 + 0.021x, respectively) as shown in Figure 2. When binning age into distinct categories of <30, 30–60, and >60 years, the linear increase in severity scoring could still be identified, irrespective of disease status. Patients over 60 years assessed nails with the highest points (7.44 ± 1.5), followed by patients between 30 and 60 years (6.85 ± 1.5) and <30 years (6.12 ± 1.7; *p* < 0.0001). These results indicate that older individuals, both psoriasis patients and healthy individuals, regard psoriatic nail changes esthetically more disturbing than younger people.

### Differences in the Subjective Assessment of Nail Severity between Subgroups of Participants

**Fig. 3:**
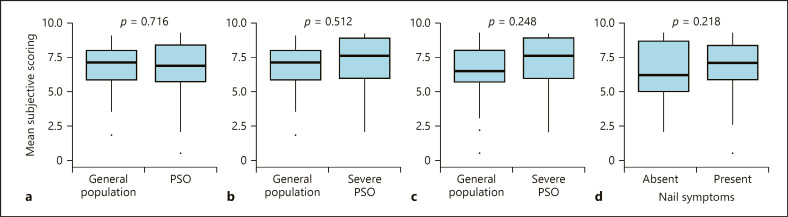
Comparison of average subjective scores of the 19 nails between different subpopulations. Boxplot of average scoring between the general population and psoriasis patients (PSO), displaying insignificant difference (**a**). A trend of higher scores are seen among psoriasis patients with more severe disease as compared to the general population (**b**) and mild psoriasis patients (**c**); however, the differences are not significant. Psoriatic patients with nail symptoms also show somewhat higher but not significantly different scores as compared to psoriasis patients without nail symptoms (**d**).

Next, we aimed to compare the subjective assessment of nails between different subgroups (patients with psoriasis and severe psoriasis vs. general population, patients with severe vs. mild psoriasis, and patients without vs. with nail psoriasis). For this, we calculated the mean subjective scores of all 19 nails of several subcategories. No difference was found between the subjective assessment of psoriasis patients and the general population shown in Figure 3a (*p* = 0.716). As a trend, patients with severe psoriasis assessed nails aesthetically more disturbing than patients with mild psoriasis or the general population; however, the differences were statistically not significant shown in Figure 3b (*p* = 0.248) and Figure 3c (*p* = 0.512), respectively. Psoriatic patients with nail symptoms also scored somewhat higher than patients without nail symptoms, but the difference was not significant, either shown in Figure 3d (*p* = 0.218).

**Fig. 4:**
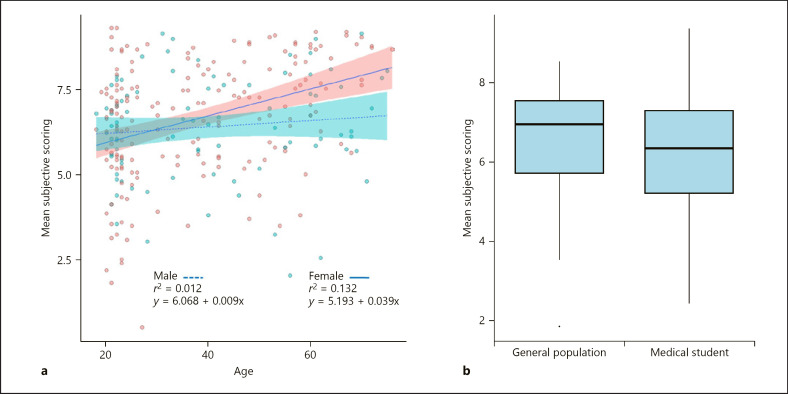
**a** Linear regression analysis of subjective nail scoring versus respondent age between sexes in the complete study cohort. As a general trend, females (red dots and red confidence interval shading, solid best fit line) show higher scoring as a function of increasing age than males (blue dots and blue confidence interval shading, dotted best fit line). **b** Boxplot of differences between medical students and participants from the general population aged<30 years. Despite controlling for age bias, medical students show a trend of lower severity scoring.

Across the complete study population, men attributed slightly lower scores to nails than women, although the difference was not significant (6.44 ± 1.5 and 6.69 ± 1.8, respectively, *p* = 0.13, and data not shown). In addition, we carried out linear regression analysis of subjective scoring and age between sexes in the complete study cohort. As a general trend, females show higher scoring than males (*r*^*2*^ = 0.132, ŷ = 5.193 + 0.039x and *r*^2^ = 0.012, ŷ = 6.068 + 0.009x, respectively), as a function of increasing age shown in Figure 4a. Thus, the general age-related increase in the subjective assessment of nail symptoms could be more pronounced in the female population.

Finally, we investigated whether professional medical knowledge influences the subjective scoring of the nail images. For this, we compared data of medical students and participants from the general population aged <30 years. Despite controlling for age bias, medical students show a trend of lower severity scoring however with no statistical difference (*p* = 0.815) shown in Figure 4b.

## Discussion

The overall aim of this study was to assess the subjective perception of psoriatic nail changes. Psoriasis frequently affects nails, leading to different degrees of functional disability in patients. It has been shown that nail disease severity correlates with skin psoriasis severity [13], functional disability [6], and higher nail psoriasis severity scores (e.g., NAPSI) are associated with more significant life quality impairment (e.g., NPQ10) [14]. Indeed, 8 of the 10 questions of the NPQ10 assess the impact of nail changes on the daily activity of the patient (the remaining two questions assess the location of nail psoriasis and the mood of the patient) [11]. Nail however is not only a functionally important part of the human integument but serves as significant esthetic constituent. Although nail changes may have important esthetic consequences, to our knowledge, our study is the first to systematically investigate esthetic perception of nail disease in psoriasis.

Based on our findings, nail symptom severity (assessed by the NAPSI score) in general correlated well with the subjective scores. Especially, nails with low (0) and high (6–8) NAPSI values received relatively consistent low and high subjective scores, respectively. This is not surprising since these nails display either very limited or very extensive clinical symptoms, generating uniform emotions in the observers. On the other hand, the esthetic perception of nails with moderate NAPSI scores was rather heterogeneous. It is likely that these differences are stemming from the inherent nature of NAPSI calculation. NAPSI is an objective and validated scoring system in nail psoriasis, although it has several limitations. Aktan et al. [15], e.g., suggested that scoring for nail bed features are more reliable than scoring for nail matrix features. Augustin and Ogilvie [16] recommended possible reconsideration of the nail psoriasis assessing system, including psoriatic patients' HRQoL parameters. Furthermore, in NAPSI, scores are given for the presence of a specific nail symptom, and the extent of the symptom is less directly proportional with the score. Pitting or splinter hemorrhage in one quadrant, e.g., leads to an equal score as complete dystrophy of the quadrant. Obviously, the esthetic perception of a few pitting symptoms or extensive dystrophy of the nail is not equal. We are fully aware that esthetical judgment is a multidimensional process, composed of respondent's own perceptual analysis and esthetic emotions toward the nail symptoms associated with psoriasis. It may include disgust, ugliness, distress, social disturbance, or other aspects as well. However, we did not define “aesthetic disturbance” further to the respondents as we wanted to determine it in a single scale. In summary, our results show that medium severity NAPSI scores do not reliably reflect the esthetic consequences of nail psoriasis.

The age of the respondent showed robust positive correlation with the subjective assessment of nail symptoms both within the psoriatic and the general population. Based on our findings, it is not clear whether this reflects the generally more optimistic view of the younger populations or a nail-specific finding. In addition to age, women scored higher than males, although the difference was not significant. Based on the findings of Ortonne et al. [11] and Klaassen et al. [17], the life quality scores are significantly influenced by gender in nail psoriasis: women have higher NPQ10 scores than men.

Interestingly, the respondents' psoriasis, severe psoriasis, or even nail psoriasis had no significant impact on the esthetic assessment of nails. Also, medical education (e.g., in the case of medical students) had no significant influence on the esthetic perception of nail psoriasis either.

In conclusion, esthetic perception of nail psoriasis does not necessarily reflect objective severity of the nails, especially in moderately severe forms of nail psoriasis. Older people consider psoriatic nail symptoms esthetically more disturbing, while gender and medical education has no significant influence on the esthetic assessment of psoriatic nail changes.

## Statement of Ethics

The authors have no ethical conflicts to disclose. All patients have given written consent to participate in this research project and to publish details of the study. This investigation was approved and controlled by the Regional Research Ethics Committee of the Medical Center, University of Pécs (Approval No. 3280/a) in accordance with the ethical principles of the Declaration of Helsinki and with Good Clinical Practice as defined by the International Conference on Harmonization.

## Conflict of Interest Statement

The authors have no conflicts of interest to declare.

## Funding Sources

This work was performed with the financial support of the Human Resources Development Operative Programme Grant (EFOP 3.6.2-16-2017-00006) of the National Research, Development and Innovation Office, Hungary, EFOP 3.6.2-16-2017-00009, EFOP 3.6.3-VEKOP-16-2017-00009, OTKA K_18_128210, and TKP-23-1/PALY-2020.

## Author Contributions

Conceptualization: Rolland Gyulai; investigation: Júlia Szebényi; methodology: Júlia Szebényi, Péter Oláh, and Rolland Gyulai; supervision: Rolland Gyulai; visualization: Péter Oláh; Writing − original draft: Júlia Szebényi and Péter Oláh; Writing − review and editing: Rolland Gyulai.

## Data Availability Statement

All data generated or analyzed during this study are included in this article. Further inquiries can be directed to the corresponding author.

## Supplementary Material

Supplementary data

## Figures and Tables

**Table 1:** Comparison of the subjective scores of the total cohort, the psoriatic patients and the general population to the NAPSI (scaled to 0–10) scores of the 19 nails (N01–N19)

Nail	NAPSI (0–10)*	Subjective score						*p* value**
		total		gen. population		psoriasis		
		median	mean ± SD	median	mean ± SD	median	mean ± SD	
N01	10	10	8.53±2.16	10	8.81±2.01	10	8.7±2.3	0.450
N02	5	9	8.44±1.95	9	8.67±1.75	10	8.62±2.14	0.252
N03	5	8	7.52±2.28	8	7.81±2.05	9	7.9±2.46	0.192
N04	0	2	2.52±2.13	2	2.77±1.98	2	2.87±2.35	0.482
N05	6	8	7.93±2.15	8	7.77±2.13	9	8.22±2.3	**0.021**
N06	10	8	7.9±2.22	8	7.96±2.01	9	8.13±2.37	0.110
N07	10	10	8.86±1.78	10	9.08±1.46	10	8.79±2.06	0.419
N08	5	4	4.19±2.58	5	4.64±2.46	4	4.29±2.67	0.186
N09	3	3	2.99±2.31	3	3.34±2.08	3	3.27±2.43	0.265
N10	6	6	5.75±2.62	6	6.21±2.43	6	6.08±2.79	0.343
N11	10	10	8.84±1.81	10	9±1.43	10	8.79±2.11	0.315
N12	3	5	5.18±2.67	6	5.84±2.5	5.5	5.29±2.92	0.075
N13	5	3	2.93±2.36	3	3.11±2.5	3	3.02±2.46	0.355
N14	6	8	7.33±2.36	8	7.36±2.28	8	7.38±2.58	0.358
N15	5	8	7.97±2.24	8	8.08±1.99	9	8.05±2.47	0.253
N16	9	8	7.23±2.33	7	7.33±2.15	8	7.25±2.57	0.359
N17	8	10	8.7±1.92	10	8.82±1.68	10	8.71±2.14	0.363
N18	8	10	9.04±1.69	10	9.16±1.43	10	8.92±1.91	0.356
N19	4	3	3.73±2.47	4	4.16±2.42	3	3.89±2.68	0.128

Comparison of the subjective scores of the total cohort, the psoriatic patients, and the general population to the NAPSI (scaled to 0–10) scores of the 19 nails (N01–N19). *p* values <0.05 were considered significant and are in bold.

* NAPSI scores (0–8) were scaled to 0–10 in order to correlate with the subjective scores of nails.

** *p* values indicate significant differences between psoriasis patients and the general population (control).

**Table 2:** Demographic characteristics of respondents

	Psoriatic patients (*n* = 106)	General population (*n* = 74)	Medical students (*n* = 86)
Age, *n* (%), years			
<30	15 (14.2)	14 (18.9)	86 (100.0)
30–60	61 (57.6)	49 (66.2)	−
>60	30 (28.3)	11 (14.9)	−
Males, *n* (%)	41 (38.7)	20 (27.0)	30 (34.5)
Nail symptoms *n* (%)	80 (75.5)	−	−
Severe psoriasis^1^, *n* (%)	30 (28.3)	−	−

1 Based on the history of anti-psoriatic drug use.
