# Supplementary material for: Comparison of the Objective Severity and the Esthetic Perception of Nail Symptoms in Psoriasis
Source: Skin Appendage Disord. 2022 Feb 14;8(4):295–301. doi: 10.1159/000521930 (PMC9275001; doi:10.1159/000521930)

**Supplementary Material 1**

Questionnaire to investigate the differences of the subjective/esthetic evaluation of nail psoriasis between psoriasis patients and the general population.

Sex:

- Male
- Female

Age: ­___

Are you a health-care worker, or a mediacal student?

- Yes
- No

Do you have psoriasis?

- Yes
- No

Questions regarding psoriasis:

Do you have psoriatc nail symptoms?

- Yes
- No

How many nails are affected by psoriasis? ___

Do you administer anti-psoriatic drug?

- Yes
- No

Please evaluate 19 nail photos subjectively - between 0 (no aesthetic disturbance) and 10 (maximal aesthetic disturbance) - based on how disturbing you consider them.

0 1 2 3 4 5 6 7 8 9 10

no aesthetic disturbance O O O O O O O O O O O maximal aesthetic disturbance

Image 1.


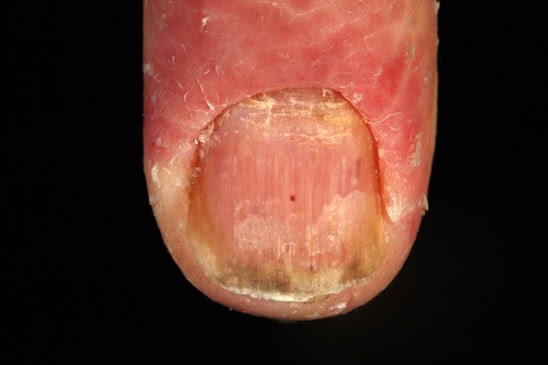


Image 2.


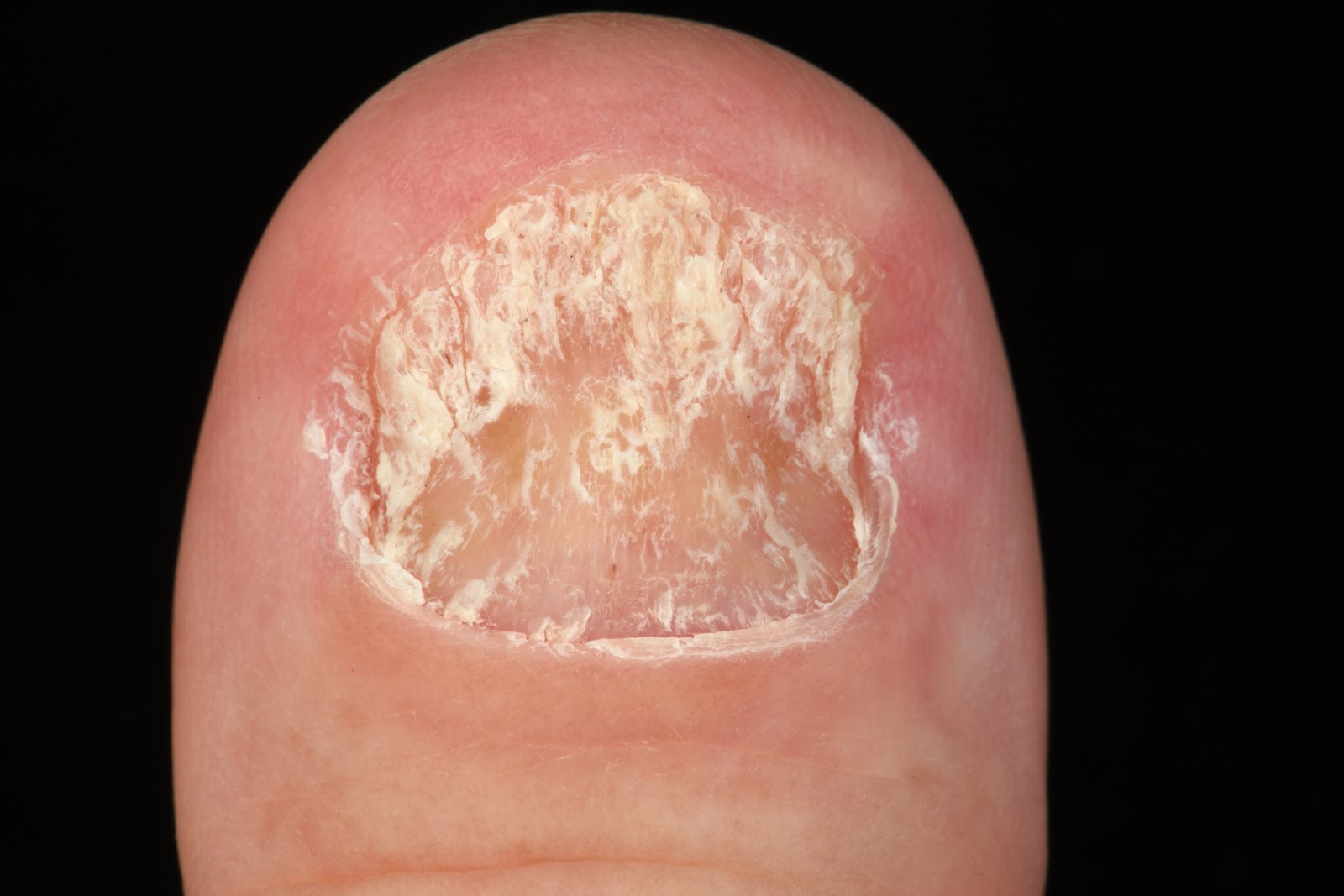


Image 3.


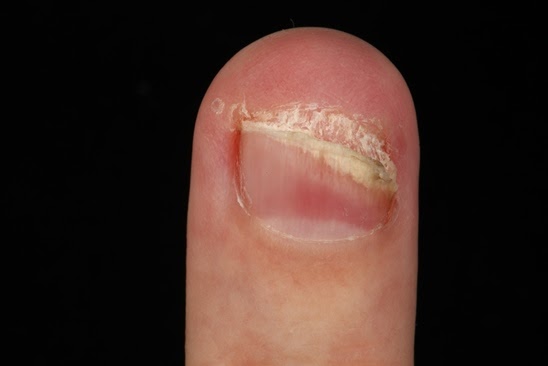


Image 4.


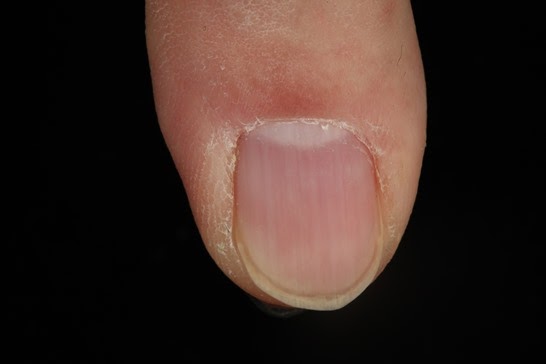


Image 5.


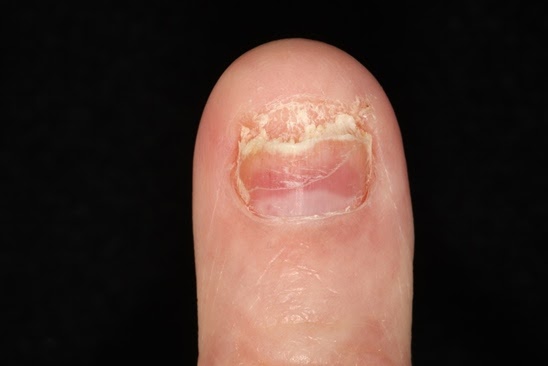


Image 6.


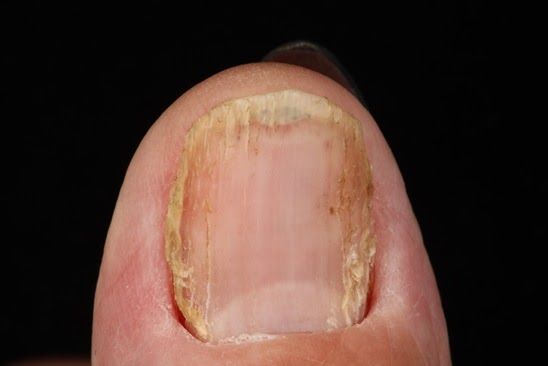


Image 7.


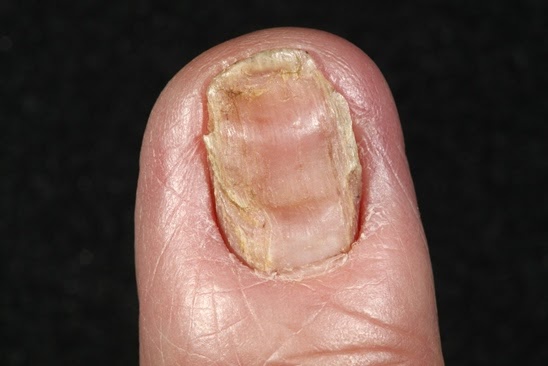


Image 8.


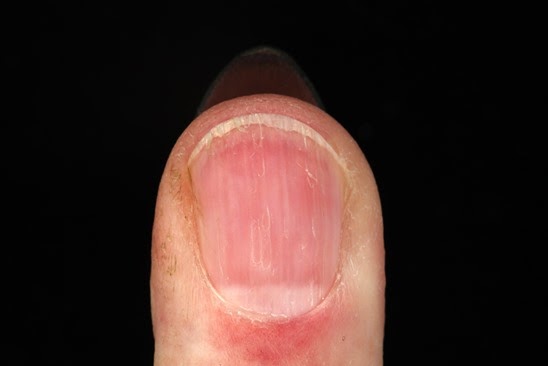


Image 9.


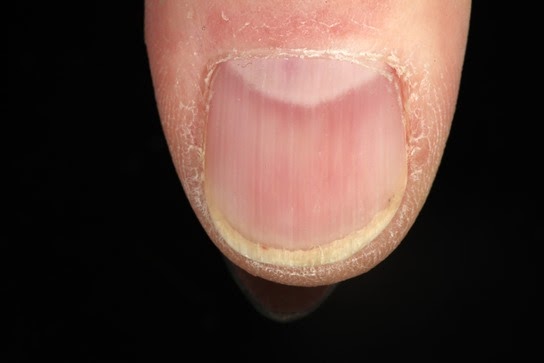


Image 10.


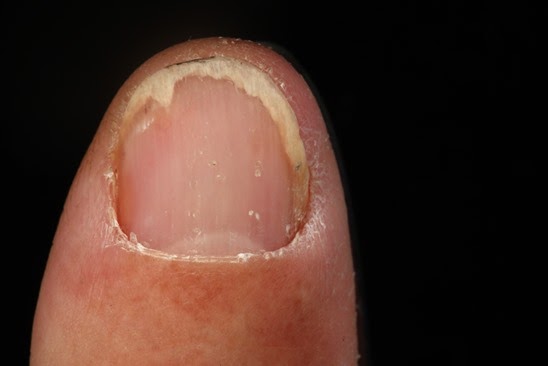


Image 11.


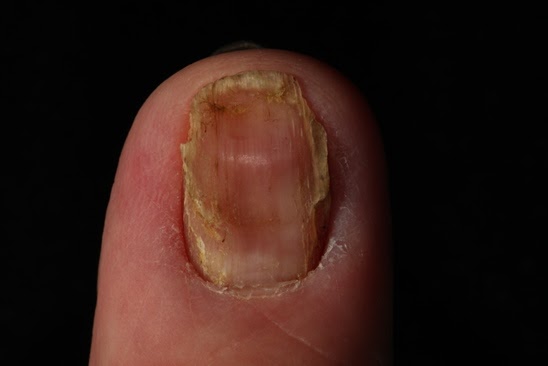


Image 12.


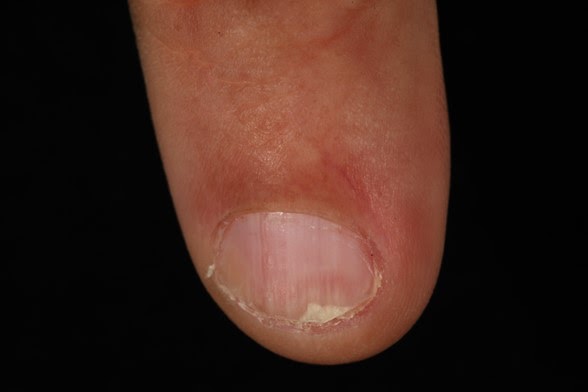


Image 13.


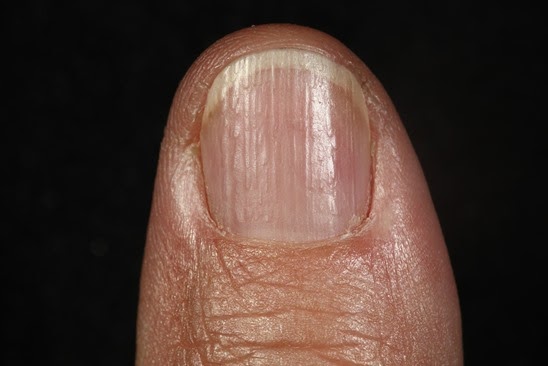


Image 14.


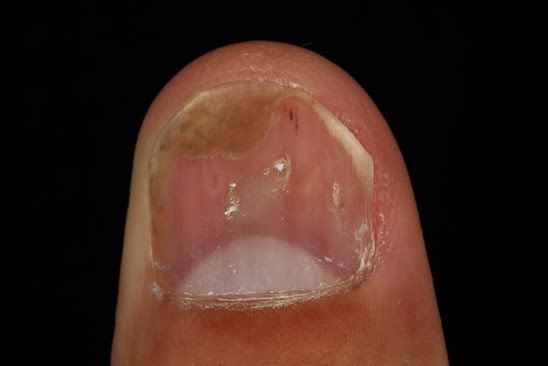


Image 15.


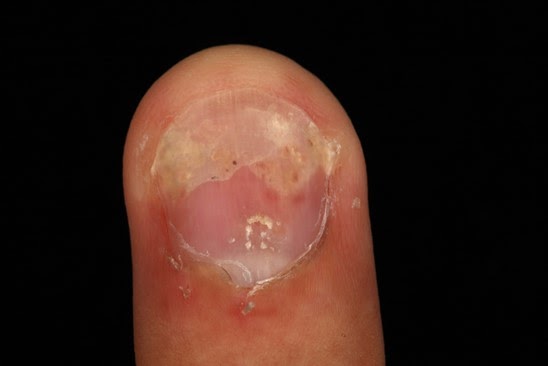


Image 16.


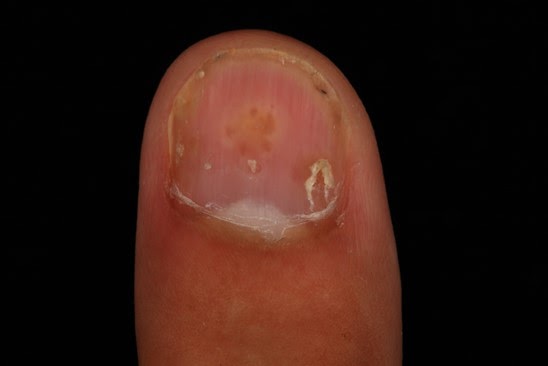


Image 17.


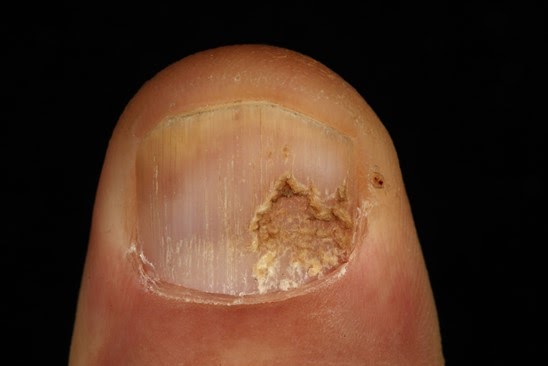


Image 18.


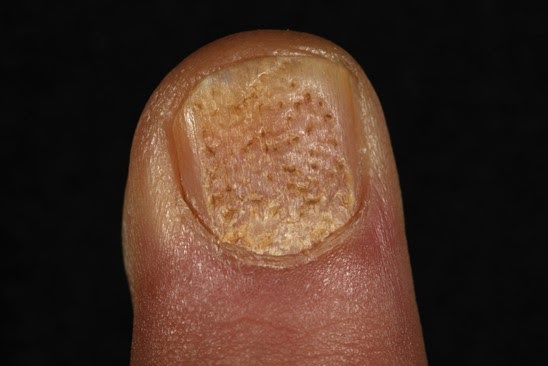


Image 19.


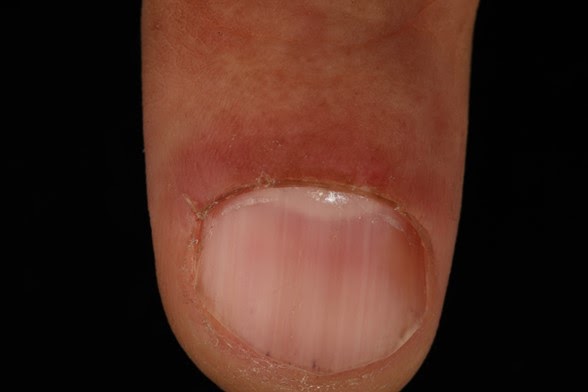

Supplement: Supplementary file 1 — Supplementary data [file sad-0008-0295-s01.docx]
